# Graphite Size Effect on Chemical Expansion and Graphene Oxide Properties

**DOI:** 10.1021/acsomega.2c05059

**Published:** 2022-10-14

**Authors:** Zineb Benzait, Levent Trabzon

**Affiliations:** † Nanoscience and Nanoengineering Department, Istanbul Technical University, Maslak, Istanbul 34469, Turkey; ‡ Department of Mechanical Engineering, Istanbul Technical University, Istanbul 34437, Turkey; § MEMS Research Center, Istanbul Technical University, Istanbul 34437, Turkey

## Abstract

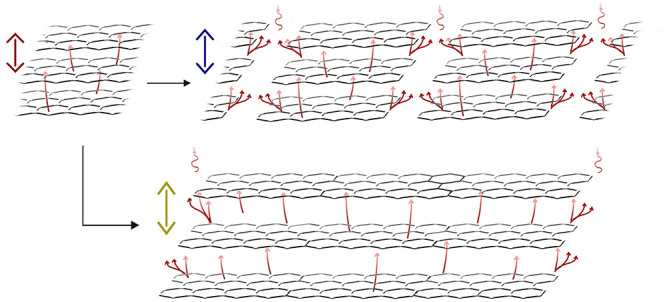

Does larger graphite flake size always lead to larger and better graphene oxide (GO)? Is there an optimum size to balance between the large building blocks needed and the defects generated during oxidation? In this study, the effect of using four different graphite flake sources on the size, structure, and properties of GO and reduced graphene oxide (rGO) was investigated. GO was mainly prepared by the enhanced synthesis method except for the smallest graphite size, which could not be expanded before oxidation. The effect of the flakes’ lateral size and thickness on the expansion volume was also studied. Several characterization techniques were performed throughout this work, and their results provide evidence of how the graphite size changes not only the expansion volume of the chemically expanded graphite (CEG) as well as the final properties of GO or rGO but also the presence of organosulfate impurities, defects, wide size distribution, and the harsh oxidation reaction itself.

## 1 Introduction

Graphene, the most known two-dimensional (2D) material due to its relatively old discovery, tunable functionalities, and wide applications, has become over the past decades one of the most emergent materials. Among its derivatives, graphene oxide (GO) is one of the most important nanomaterials due to its many advantages such as being solution processable, feasibility of being produced on a relatively large scale, and having many commercialization prospects.^1^ Among its wide applications, GO was demonstrated to be attractive in membranes,^2^ polymer nanocomposites for barrier coatings^3^ and reinforced films and fibers,^4,5^ composite electrodes for batteries and supercapacitors,^6−8^ biosensing and diverse biomedical applications,^9,10^ and photonics.^11^

It is more convenient in most of these applications to have a big aspect ratio, and thus, a large lateral size of GO or reduced graphene oxide (rGO) monolayers: for example, unlike those made of large sheets, membranes assembled from small GO sheets have leaking paths due to boundaries and low mechanical properties.^12^ Another example is given by a study on a flexible graphene paper that showed exceptional electromagnetic interference (EMI) shielding performance thanks to using large rGO sheets, which ensure better alignment.^13^ Large graphene sheets could also help adjust the characteristics of localized surface plasmons (LSPs)^14^ and increase the surface area and conductivity of electrodes.^15^ Even at the level of functionalized-graphene quantum dots (F-GQDs), the physical mechanism of their photoluminescence (PL) emission is related to their size, where the surface/edge state including oxygen groups that causes twist geometries plays an important role, and where the absorption peaks red-shift progressively with the increase of size, which is attributed to the enhanced π-electron delocalization with increasing conjugation length, resulting in a reduced electron–phonon coupling strength.^16,17^

However, the production of large GO (LGO) is challenging, generally due to harsh reaction conditions (high temperature, excessive oxidant quantity, etc.), crack formation throughout the cross-planar oxidation, and crack formation throughout the agitation or sonication.^18−20^ Several approaches have been used in order to produce LGO, such as their separation via centrifugation,^21^ use of expanded graphite,^22,23^ use of low reaction temperature,^24^ avoidance of stirring during graphite swelling,^20^ change of the starting material type,^25^ and use of initially large graphite flakes.^26,27^

In our previous work,^23^ we prepared LGO by a method called the “enhanced synthesis method”, where firstly we did a conventional pretreatment of graphite flakes to obtain expanded graphite by an environmentally friendly one-step approach using only piranha solution. We combined it with reducing the reaction temperature and the stirring frequency while keeping the same reactant quantity and washing steps as described by Tour et al.^28,29^ In the present work, we wish to further integrate it with using large graphite flakes as a starting material, while making a few further enhancements like reducing the acid quantity to reduce the total cost and make it more environmentally friendly,^30^ performing it at room temperature, and minimizing the oxidant quantity to avoid any overoxidation that can induce more defects that are hardly removed through reduction.^19,31^

When increasing the initial graphite flake size, one can assume that GO size will always increase proportionally, and thus the final product properties. However, this may not always be true, there may be an optimum size where the large building blocks needed and the defects generated are balanced because larger graphite flakes need a higher oxidant quantity to overcome the diffusion-controlled oxidation pathways until arriving at the flake’s middle, which may cause harsh oxidation and create more cracks and defects. To verify this hypothesis, four natural graphite sources having different sizes were used in the current study, and their influence on the physicochemical properties of GO single sheets as well as freestanding films was studied.

In fact, there are few studies on the effect of parent graphite on GO. Peng et al.^32^ studied the electrochemical performance of rGOs prepared from natural flaky, lumpy, and amorphous graphites using Hummers’ method. Jasim et al.^25^ synthesized few-layered GO sheets from flakes, then ground and powdered them for a biological application. Botas et al.^33^ used a synthetic graphite from a mixture of polycyclic aromatic hydrocarbons; the texture and crystal size were varied, and the maximum particle diameter of the two obtained synthetic graphites was 80 μm. Shojaeenezhad et al.^34^ used clod powder with two different sizes of 18 and 6 μm. Nevertheless, in the present work, we focused on using parent graphite of four groups of natural flakes with a size of up to 660 μm. The aim was to make the protocol more economical (natural flakes) and at the same time obtain the large GO needed for many applications, especially the ones that need high mechanical properties. Dao et al.^35^ studied how the size of purchased and expanded graphite affects the oxidation degree and chemical structure of GO. However, in the present work, our previous enhanced synthesis method was used to prepare GO. Unlike in the cited works, we tried to obtain a similar oxidation level to eliminate its effect on the final mechanical and electrical properties; at the same time, we tried to minimize the oxidant quantity required for each graphite size to avoid any overoxidation that creates more defects.

The results show that the best GO freestanding film was obtained from the graphite with the largest flakes and had 232 ± 11 MPa strength, 11.3 ± 1.6 MJ m^–3^ toughness, and ∼500 S cm^–1^ conductivity upon reduction. Yet, larger graphite flake size does not always lead to larger and better GO, and does not have the same proportionality. Impurities, defect degree, the harsh oxidation reaction itself, and many other parameters may strongly affect the properties of the final material.

## 2 Materials and Methods

In order to select the suitable starting material that can yield GO with the best properties, several sources of natural graphite flakes that have different sizes and have been widely reported in literature^20,26,28,36^ were used: 325 mesh (i.e., around 44 μm) and 200 mesh (i.e., around 74 μm) from Qingdao Huatai Lubricant Sealing S&T Co., Ltd. +100 mesh (≥75% larger than 150 μm) from Sigma-Aldrich cat #332461, and +50 mesh (≥80% larger than 300 μm) from Asbury Graphite Mill inc cat #3061.

Potassium permanganate (KMnO_4_) with a purity higher than 99% was obtained from NeoFroxx (LC-7081). Sulfuric acid (H_2_SO_4_) 95–97% was obtained from Merck, phosphoric acid (H_3_PO_4_) 85% and hydrogen peroxide (H_2_O_2_) 30% were purchased from Carlo Erba, and finally, hydroiodic acid (HI) for analysis in 57 wt % stabilized aqueous solution was obtained from Acros (19837).

### 2.1 Expanded Graphite (EG) Preparation

For the expansion of +50 and +100-mesh graphite flakes, the same method reported in Benzait et al.^23^ was used. Fifty millilters of a fresh piranha solution (9:1 of H_2_SO_4_/H_2_O_2_) was prepared and cooled using an ice-water bath. 0.5 g of graphite was added and stirred for 10 min at 300 rpm. The magnetic rod was then removed and the mixture was left overnight (16 h) at ∼30 °C.

For the expansion of 200-mesh graphite flakes, the ratio of piranha solution to graphite mentioned above (100 mL to each 1 g graphite) did not lead to a high expansion, and further, they remained apart from the liquid. In this case, the oxidation did not completely occur due to the remaining H_2_O_2_ that reacts with the oxidant, thereby inhibiting the oxidation. Thus, the piranha solution volume was optimized to 40 mL for each 1 g of 200-mesh graphite.

### 2.2 GO_325_ Synthesis

The 325-mesh graphite did not expand despite trying different piranha solution volumes; thus, graphene oxide GO_325_ was obtained through direct oxidation of the 325-mesh graphite as follows: 0.5 g of graphite, 20 mL of 9:1 acidic solution of 18 mL of H_2_SO_4_, and 2 mL of H_3_PO_4_ were stirred at room temperature (20 °C). After 30 min, 1.5 g of the oxidant (KMnO_4_) was added slowly to avoid becoming explosive.^29^ The reaction mixture was then kept at room temperature under magnetic stirring (300 rpm) for an oxidation time of 6 h.

### 2.3 Enhanced GO_200_, GO_+100_, and GO_+50_ Synthesis

**Figure 1:**
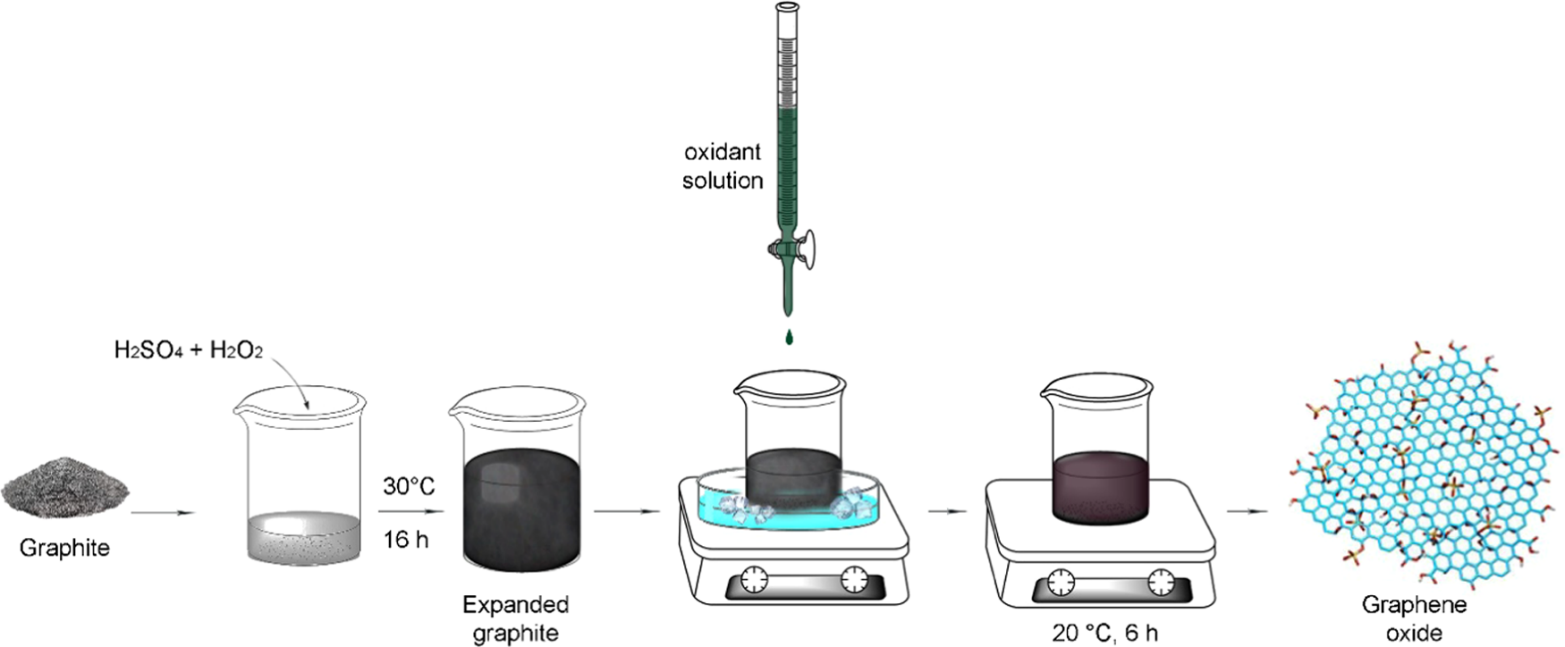
Schematic illustration of the synthesis of GO by the enhanced method with slight modifications.

The graphene oxide samples were prepared from their EG according to the enhanced method^23^ with slight modifications (Figure 1) such as using fewer acids (40 mL per 1 g graphite) to make it more cost-effective and more environmentally friendly,^30^ performing it at room temperature to make it more practical, and minimizing the oxidant quantity (4, 6, and 7 g per 1 g of graphite) required for each graphite size (200, +100, and +50 mesh, respectively) to avoid any overoxidation.^19,31^

First, a 9:1 mixture of concentrated H_2_SO_4_/H_3_PO_4_ (18 mL:2 mL) was cooled using an ice-water bath, and a given oxidant (KMnO_4_) quantity was added and stirred at 500 rpm for 30 min. This cold oxidant solution was slowly added (∼10 min) to the EG prepared from 0.5 g of each graphite size; the addition was done while stirring the mixture mechanically and keeping the bath temperature lower than 10 °C. After the complete addition, this mixture was left at room temperature and stirred each half an hour during the first two hours.

After the oxidation time, which was fixed at 6 h, each reaction was stopped by adding ∼200 mL of ice cubes to promote better temperature control, and ∼1 mL of H_2_O_2_ in order to decompose the insoluble manganese salts.^26^ Few unreacted black particles were found floating above the acidic solution, and were removed using a plastic pipette. DI water was added to the mixture, which was left overnight. The color of the three solutions is almost identical (Figure S1), indicating a similar oxidation degree of the resulting GO. We tried to obtain a similar oxidation level to eliminate its effect on the final mechanical and electrical properties. Lower quantities of KMnO_4_ led to low oxidation and exfoliation, manifested by the gray-brown color (examples in Figure S2a–c), while higher quantities led to more brownish-yellowish solutions (Figure S2d), indicating an overoxidation.

### 2.4 GO Purification

GO was found spontaneously sedimented; thus, it could be easily separated from the acidic solution. To speed up the purification process, each GO was washed by dispersion and centrifugation (Nüve NF 800) at 8000 rpm with 50 mL of HCl 37% (diluted with DI) and then repetitively with DI water until achieving a pH of ∼6. Note that after each centrifugation and re-dispersion cycle, the GO got more swelled and a longer centrifugation time was needed (from 5 to 30 min) to sediment the GO sheets. Each GO aqueous solution was further exfoliated by magnetic stirring at 300 rpm for 5 min and bath sonication at ∼74 W/L for 10 min. In order to remove any remaining black particles or unexfoliated sheets, 4 cycles of low-speed centrifugation at 4000 and 2000 rpm were run; the sediments were then discarded and the supernatants were collected as the final purified GO solutions.

### 2.5 Freestanding Films’ Preparation and Reduction

The GO solutions were concentrated through 4 h of centrifugation at 9000 rpm to obtain viscous solutions, which were made more viscous by heat treatment at 70 °C for 36 h, and then coated on PTFE substrates using a film applicator. The obtained films were dried overnight at room temperature, peeled off easily from the PTFE substrates, and stored in a desiccator for around two days. The film applicator thickness was controlled so that the final films’ thicknesses are in the range of 5.5–7 μm.

In order to obtain rGO films for the conductivity measurements, 20 mm × 10 mm strips of the freestanding GO films were cut and submerged in 57% hydroiodic (HI) acid in small Petri dishes for 3 h at room temperature, then thoroughly washed, dried, and stored in a desiccator.

### 2.6 Characterization

For tensile tests, GO freestanding films were cut into sizes of 3 × 32 and prepared for being tested using a Zwick/Roell Z0.5 universal machine as described before.^23^ The width of each tested strip was measured using a Mitutoyo Absolute micrometer with a resolution of 0.01 mm, and the thickness was measured using a Mitutoyo 293-100-10 micrometer with a resolution of 0.1 μm. The average mechanical properties for each film and their standard deviations were calculated from at least 3 sample strips.

The electrical conductivity values of the rGO films were determined by the relation *C* (*S*/*m*) = 1/(*R* × *w*), where *R* is the average square resistance (unit: ohms/sq) of each sample measured using an M-3 handheld four-point probe tester at room temperature and corrected by a correction factor that depends on the samples’ shape, and *w* is the average thickness. The *R* values are taken when the measurements gave stable values.

For morphological characterization, scanning electron microscopy (SEM) of the graphite flakes was carried out with TESCAN VEGA 3. Raman analysis was performed using a RENISHAW inVia spectrometer with 532 nm excitation laser, 1% laser power, 10 s acquisition time, and x50 objective lens. For spectral analysis, the peaks were fitted by Lorentz peak function using OriginPro2021b.

For chemical characterization, the concentrations of the GO solutions were determined by filtering a certain volume of the purified solutions using a vacuum-assisted filtration (VAF) set with ∼0.22 μm PTFE hydrophilic membranes (Haining Yibo, China) and weighing the resulting GO films. The required quantity of each GO solution was prepared by serial dilutions to perform UV/visible analysis using a Scinco Neosys 2000 spectrophotometer.

∼5 μg/mL GO solutions were prepared by diluting the previous solutions, then drop cast on 300-nm-thick SiO_2_/Si substrates to visualize the sheets. The substrates were pretreated by oxygen plasma to make them hydrophilic and help spread each solution drop. To enhance the visibility of GO sheets under an optical microscope (OM) without the need of a scanning electron microscope (SEM), a few drops of hydroiodic acid were dropped onto each dried sample and left for a few minutes to allow GO reduction under room temperature, then washed and dried under a lab fume hood. GO freestanding films were directly characterized using a Perkin Elmer UATR Two Fourier transform infrared (FTIR) spectrometer and a SPECS FlexPS X-ray photoelectron spectroscope (XPS).

## 3 Results and Discussion

First, the mechanical and electrical properties of the freestanding films were determined, and then, a detailed characterization of the different graphite, GO, and rGO materials was done in order to understand the reason behind the properties’ differences observed.

### 3.1 Mechanical and Electrical Properties

**Figure 2:**
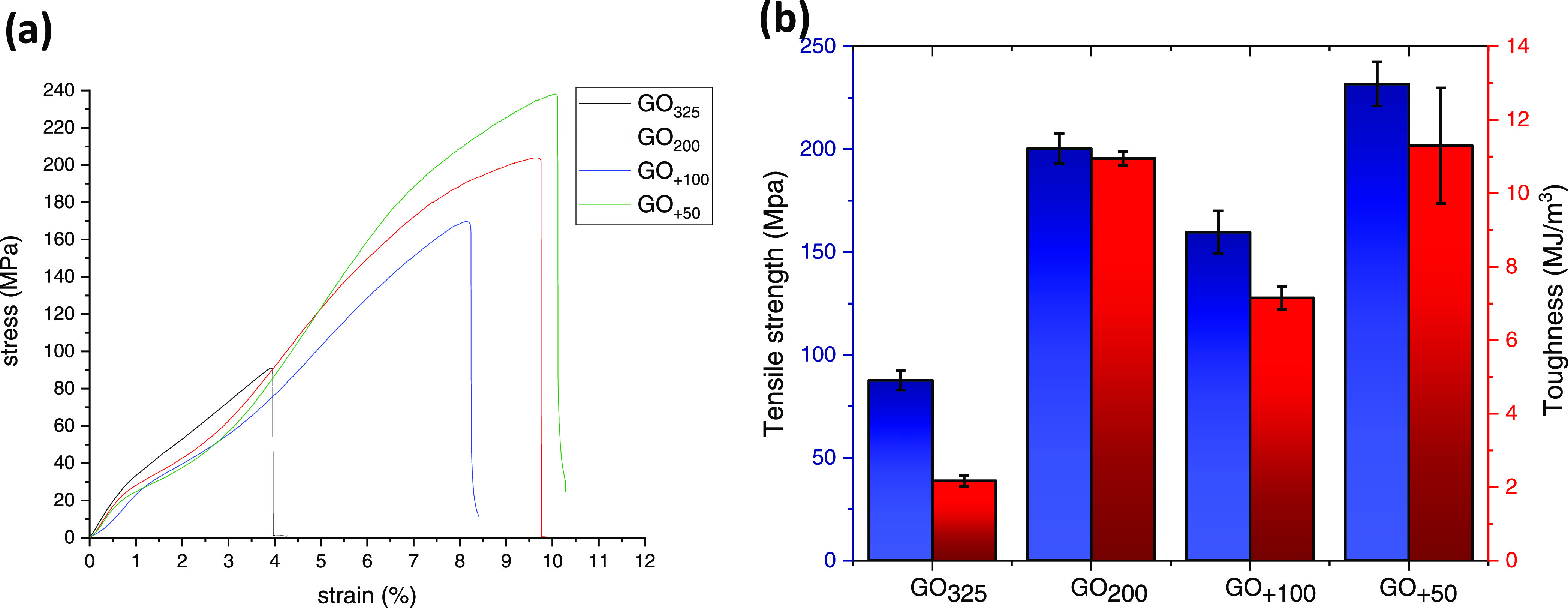
(a) Tensile curves of various GO films undergoing mechanical test. (b) Comparison of their tensile strength and toughness.

Figure 2a shows the samples’ stress-strain curves, which look nearly identical in shape but different in mechanical properties as extracted and illustrated in the column graph (Figure 2b). GO_325_ produced from 325-mesh graphite flakes—intensively used by many researchers—performed the worst and achieved only 88 ± 5 MPa of strength and 2.2 ± 0.2 MJ/m^3^ of toughness. In a general view, the strength and toughness increased with the increase of the starting graphite material size, except GO_+100_, which was unexpectedly inferior to GO_200_. In this study, GO_+50_ exhibited the highest failure strength and toughness at 232 ± 11 MPa and 11.3 ± 1.6 MJ/m^3^, respectively, but despite that its starting material had a much larger size than that of GO_200_, the difference between their tensile curves was not that pronounced. A similar tendency was noticed in the work of Perumal et al.,^27^ where the super capacitance of GO obtained from different graphite sizes was compared.

GO films were further reduced with HI acid, aiming to determine their electrical conductivities, which were found to be equal to ∼388, 536, 406, and 499 S/cm, respectively. Here, rGO_325_ revealed again the lowest value, and rGO_+100_ was again inferior to rGO_200_, while rGO_+50_ has a conductivity close to that of rGO_200_. To understand the factors that led to the results above, further characterization of the films and their starting materials was carried out.

### 3.2 Graphite and EG Characterization

**Figure 3:**
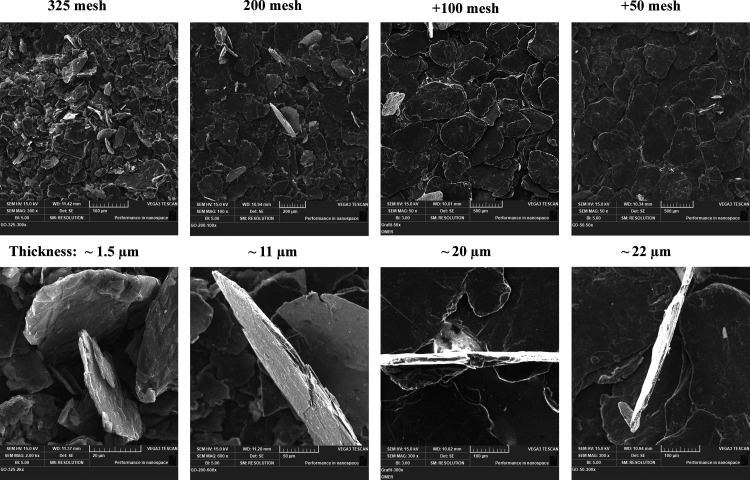
SEM of pristine graphite flakes.

The pristine graphites from different sources (Figure 3) all have a flake-like morphology. The flakes’ lateral sizes of the 325-mesh graphite vary from ∼10 to 80 μm, of the 200-mesh graphite from ∼30 to 300 μm, of the +100-mesh graphite from ∼120 to 620 μm, and finally, of the +50-mesh graphite from ∼140 to 660 μm. Compared to the +100-mesh graphite, the +50-mesh graphite has a narrower distribution: while the +100-mesh graphite is ≥75% larger than the 100-mesh size, i.e., 150 μm according to the manufacturer’s datasheet, the +50-mesh graphite is ≥80% larger than the 50-mesh size, i.e., 300 μm, and almost all of the −30-mesh graphite, i.e., ≤600 μm, according to the manufacturer’s datasheet. The enlarged SEM image of a vertical flake of each graphite shows that their thicknesses are around 1.5, 11, 20, and 22 μm, respectively.

**Figure 4:**
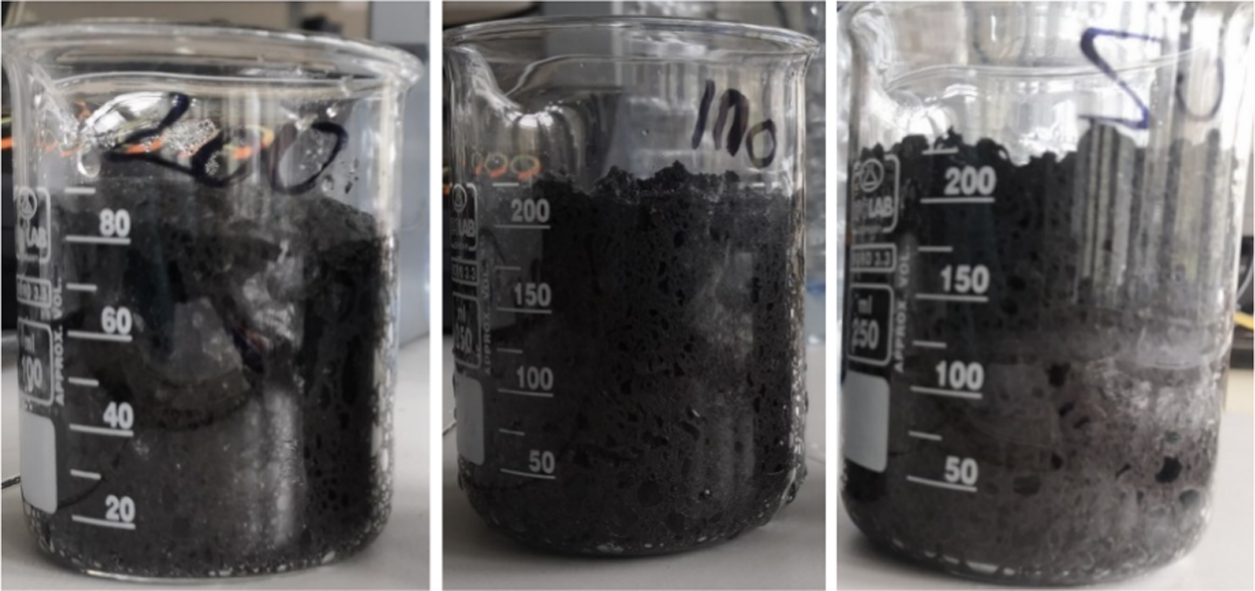
Expansion volume of expanded graphite (85, 215, and 225 cm^3^ by the initial 0.5 g of 200-, +100-, and +50-mesh graphite).

**Figure 5:**
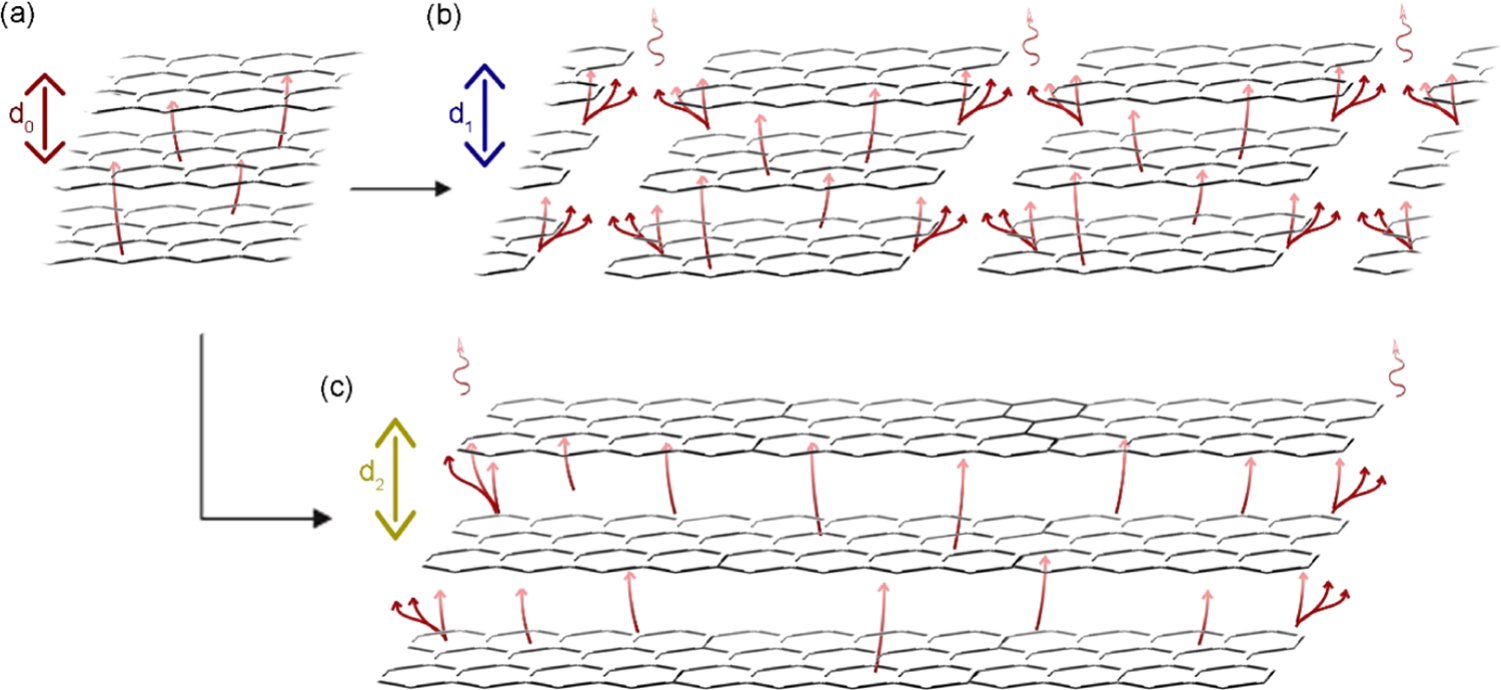
Schematic of pathways for intercalant gas (a) initially, (b) after mostly being escaped in small flakes, and (c) when being mostly trapped between layers in large flakes (*d*_0_ < *d*_1_ < *d*_2_).

Exempting the smallest and thinnest flakes of the 325-mesh graphite, the compacted structure of the other graphite flakes could be expanded by the Piranha solution, giving an expansion volume of around 170, 430, and 450 cm^3^/g from the 200-, +100-, and +50-mesh graphite, respectively (Figure 4). This result can be explained as follows: the expansion could depend strongly on the flake sizes and thicknesses:

The smaller the flakes, the more the pathways for the intercalant gas to escape in between, instead of entering between each flake’s layers, resulting in less pressure needed to push the adjacent graphene layers apart, and thus, a low expansion volume is obtained. On the other hand, the larger the flakes, the more the trapped gas between the layers, building up much pressure that separates each flake’s layers and causes—in total—a higher expansion volume or ratio, as illustrated by the simplified schematic in Figure 5. A detailed explanation of the efficiency of the intercalation process as a function of the flake dimensions can be found here.^37^

The proportional dependence of the expansion volume on the flakes’ thickness is more obvious as it results from the interlayer spacing multiplied by the number of layers. Consequently, the thicker the flake, the higher the number of layers, and the larger the total expansion volume. The highest expansion volume obtained in our study is ∼450 cm^3^/g from +50 mesh graphite which is composed of the largest and the thickest initial flakes.

**Figure 6:**
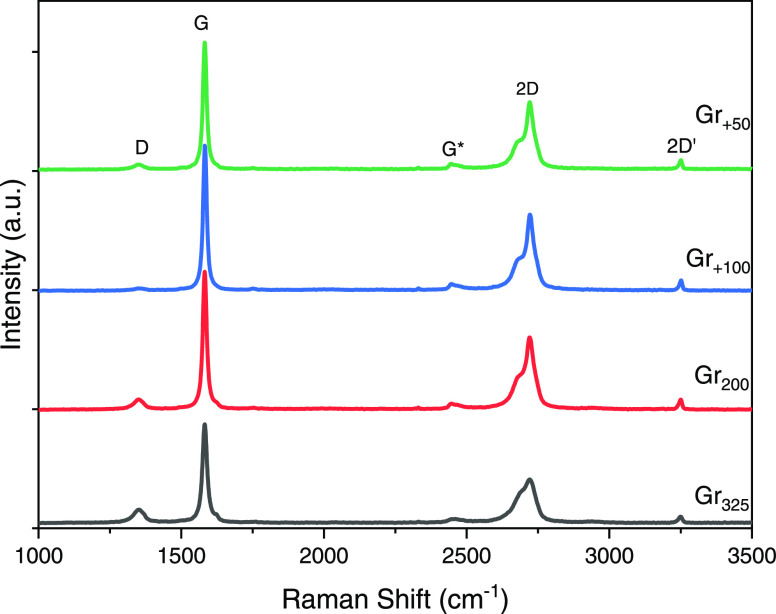
Raman spectra of graphite specimens.

Raman spectroscopy is a valuable tool often used to study graphene-based materials. The spectra of the four graphite specimens are displayed in Figure 6. As we discussed earlier, graphite consists of sp^2^-bonded planar graphene layers stacked via Van der Waals weak interactions. Thus, its spectra exhibit mainly G mode around 1580 cm^–1^ and the 2D (called by some: G′) mode around 2700 cm^–1^ related to the sp^2^-hybridized carbon bonds in the graphene lattice.^38,39^

The 2D band has a two-peak profile: the first as a shoulder called 2D_1_ band and the intense at high energy called 2D_2_ band. This splitting is understood by reason of the splitting of π electron dispersion energies triggered by the interaction between the neighboring graphitic planes.^40,41^ 2D_1_ and 2D_2_ peaks are approximately 1/4 and 1/2 of the height of the G peak, respectively; and the deconvolution of the 2D main peak into these two sub-peaks is presented in Figure S3. It is known that the increase in the number of layers induces a relative increment of the intensity of the higher frequency 2D_2_ compared to the 2D_1_ sub-peak.^42^ Hence, the intensities ratios of these sub-peaks I(2D_2_/2D_1_) are calculated and reported in Table S1. As expected, this ratio increased from Gr_325_ to Gr_+50_ in agreement with SEM thickness measurements.

Other tiny peaks like D at ∼1350 cm^–1^, G* at ∼2450 cm^–1^, and 2D′ at ∼3200 cm^–1^ are also present in these graphite specimens’ spectra and are ascribed to structural imperfections which could be disorder or defects like edges, dislocations, cracks, dopes or vacancies in the samples.^39^ G* and 2D′ modes have the same intensities in all spectra, while the D peak is considerably small in Gr_200_, almost absent in graphite with large flakes (Gr_+100_ and Gr_+50_) which signifies their well-ordered structure^38^ but cannot be negligible in Gr_325_ sample. Besides, as the disorder increases, the 2D_1_ shoulder shift upwards^41^ and becomes much closer to the 2D_2_ band in the sample Gr_325_ (Table S1). So more than its small size, these disorders and defects in the starting material of GO_325_ may be one of the reasons for its low mechanical and electrical properties reported above.

### 3.3 GO and rGO Characterization

As a 2D material, the different sizes of GO single sheets significantly affect the mechanical and electrical properties of the final bulk material as freestanding films. Generally, the size of these sheets is limited due to their fragmentation along many lines during the graphite or expanded graphite’s chemical oxidation.

**Figure 7:**
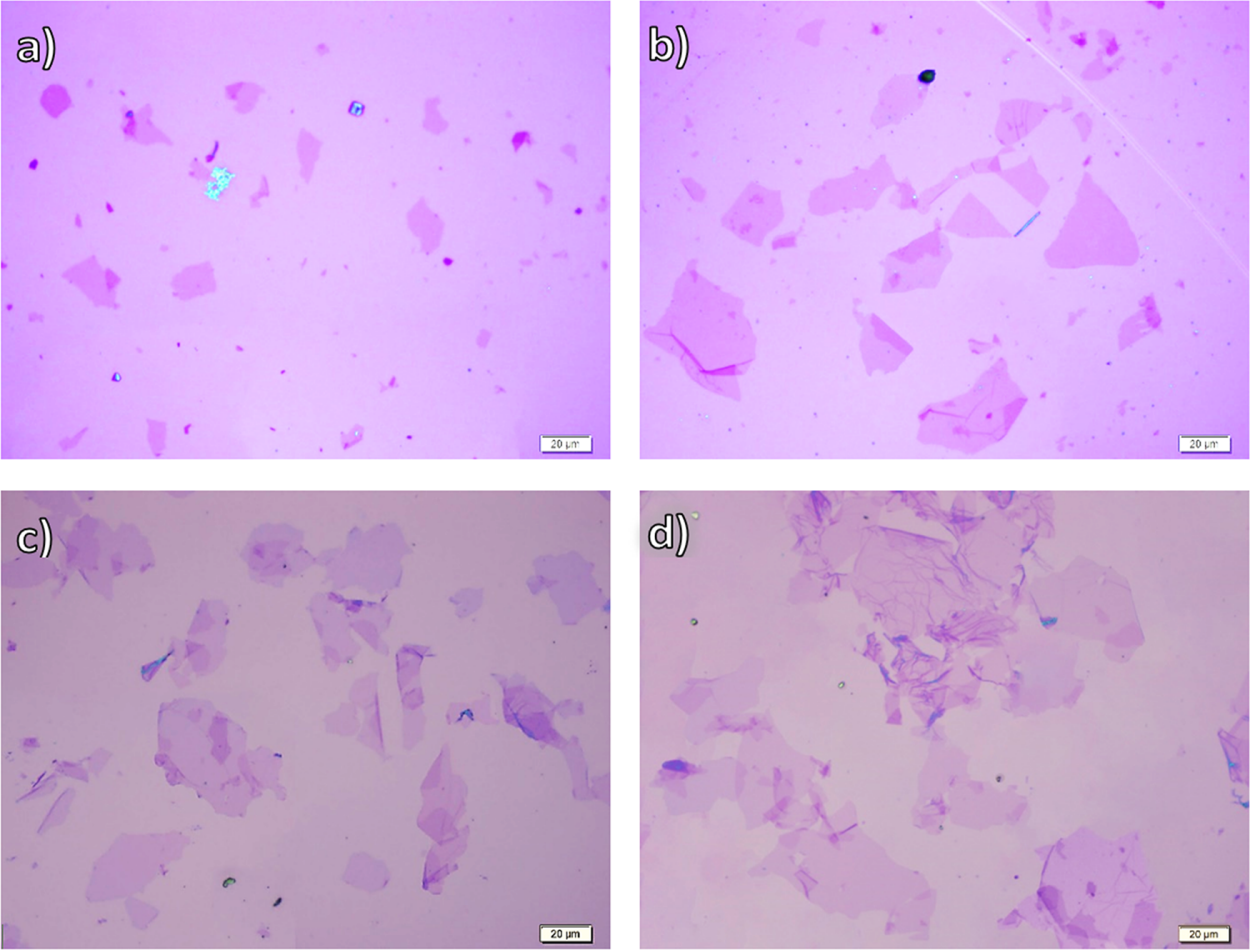
Optical microscope images of (a) GO_325_, (b) GO_200_, (c) GO_+100_, and (d) GO_+50_ (reduced on wafer).

The optical images of highly diluted GO dispersions from different raw graphite sources are shown in Figure 7 after their drop casting on Si/SiO_2_ substrates and subsequent HI reduction and washing to obtain clear images. ImageJ was used to measure the area of the sheets; we assume that each sheet is a quadrangle,^22^ with the side length of *x* and thus a sheet area of *x*^2^. In a unit interval (0–5 μm, 5–10 μm, ···etc.), the number of sheets is counted and their statistical analysis is performed using OriginPro and is illustrated in Figure S4. All of the sheet sizes got reduced multiple times from the initial graphite flake sizes due to the oxidation reaction as well as due to external energy input such as shaking during purification cycles and during the sample preparation, adding to that the sonication.^18−20^

Visibly, the GO_325_ sample sheets have the smallest sheet sizes (7.5 ± 5.2 μm, Table S2) with a wide distribution, but they are almost all less than 20 μm. No obvious difference between GO_200_ and GO_+100_ samples is observed, and their mean size is around 14 μm. The GO_+50_ sample has clearly some ultra-large sheets (≥50 μm), but its histogram reveals also a wide size distribution. All of these results would closely relate to the original dimension of the graphite flakes, and to the expansion volume. As demonstrated in our previous study,^23^ the expansion of graphite before its oxidation reduces the harsh oxidation, and thus the fragmentation of the sheets; however, the GO_+100_ sample produced from large flakes compared to GO_200_ had similar monolayer sizes, i.e., didn’t show the same trend of sheet size after expansion and oxidation. This could be attributed to the possible existence of a high number of grain boundaries and Stone–Wales defects,^31^ or to some rearrangement defects, as will be shown in the following Raman results.

GO_+50_ exhibited indeed larger sheet sizes compared to GO_200_, but not as large as we expected. This reminds us of the comparison between their mechanical and electrical properties made in the beginning, and how the difference is not proportional to the difference in their graphite flake sizes but may be attributed to their GO sheet sizes. The unexpected sizes of both GO_+100_ and GO_+50_ could be due to the cross-planar oxidation pathways of graphite from the (002) plane, which cause some periodic cracks of the uppermost GO layers and limit their sizes.^18^

**Figure 8:**
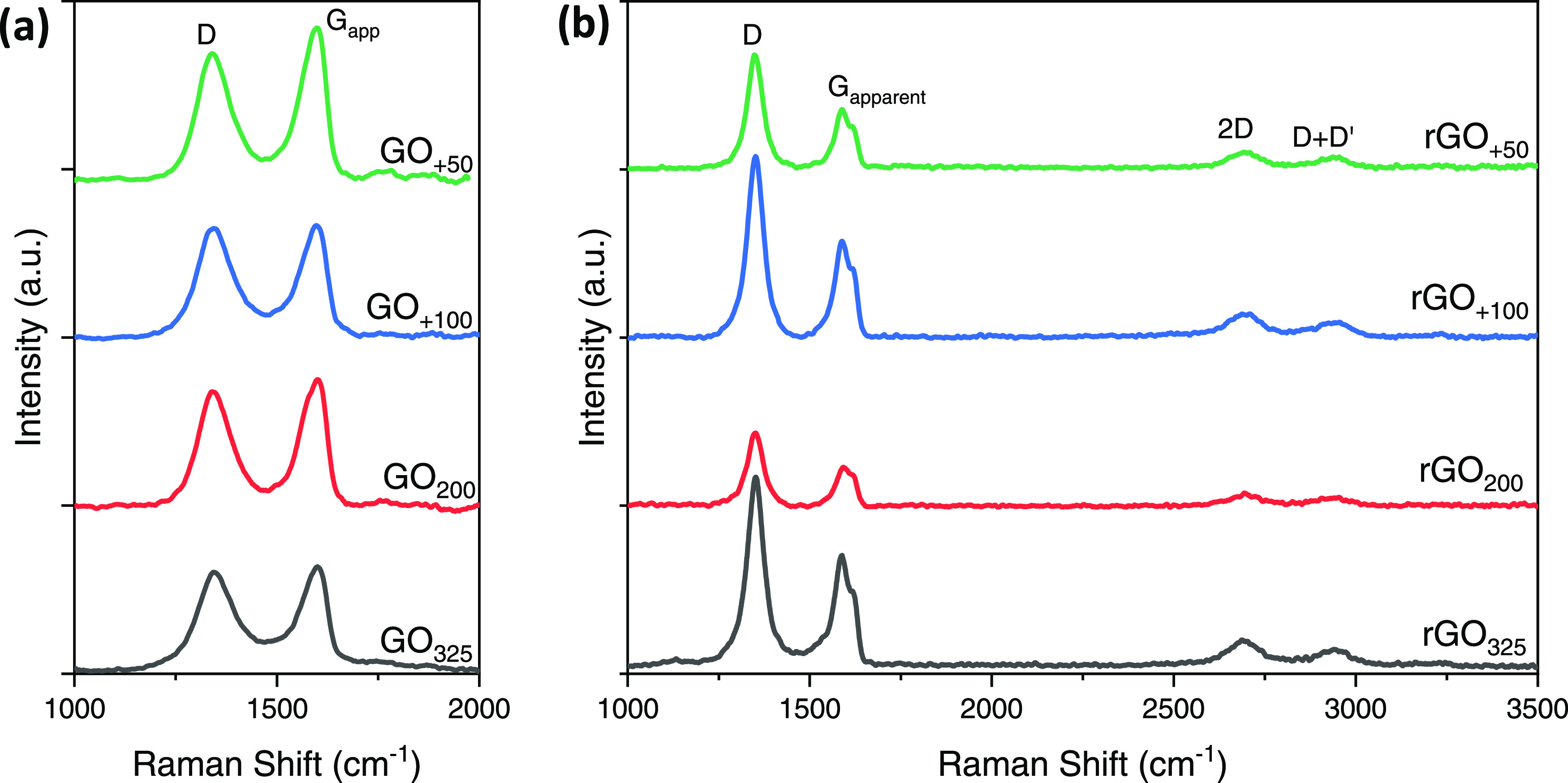
Raman spectra of (a) GO and (b) rGO films.

Raman spectroscopy is used again here to probe the structural defects on the freestanding GO and rGO films, which contain thousands of sheets and thus may be more representative of the produced materials. All of the spectra shown in Figure 8 contain mainly 3 peaks at ∼1350, ∼1588, and ∼2690 cm^–1^, which are D, G_apparent_, and 2D peaks. G_apparent_ is further deconvoluted as illustrated in Figures S5 and S6 into D** (1500–1540 cm^–1^), G (1570–1590 cm^–1^), and D′ (1605–1620 cm^–1^) peaks.^41,43,44^

As already mentioned, the G and 2D peaks are characteristic of sp^2^ hybridized carbon–carbon bonds, and are strongly present in graphite, graphene, GO, and rGO; however, the 2D band is more sensitive to defects; thus, it disappears or decreases a lot in GO and rGO dominated by a fully or partially disordered sp^2^ bonding network.^39^ On the other hand, D, D**, and D′ are bands that can be observed only when there are defects in the analyzed materials.^45^

Many researchers use the intensity ratio of the D and G_apparent_ band (*I*_D_/*I*_G-app_) as an indirect estimation of the disorder level in GO. In our case, the order of this ratio is GO_+100_ > GO_325_ > GO_200_ > GO_+50_ (Table S3), i.e., it decreases on increasing the initial graphite size, except for GO_+100_, as manifested in the highest *I*_D_/*I*_G-app_ ratio, which can be linked to its relatively small fragmented sheet size, as well as to the relatively low mechanical and electrical properties obtained for this particular material.

Vecera et al.^46^ found that the intensity values of both *I*_D_ and *I*_G-app_ peaks increase with the degree of functionalization of the graphene derivatives. In our case, the D and G_app_ peaks in GO_200_, GO_+100_, and GO_+50_ are more intense than those in GO_325_; besides, they have close intensity values, possibly due to their similar oxidation degrees. Furthermore, compared to GO_325_, they have a higher ratio of the sum of areas under D, D**, and D′ peaks over the G peak (*A*_(all D peaks)_/*A*_G_), probably due to possessing a higher density of on-plane functionalization defects of graphene sheets.^31^ Another remarkable result is the presence of a large D** peak in GO_325_ (*A*_D**_ = 7.6) followed by GO_+100_ (*A*_D**_ = 5.4); this peak has been often related to the amorphous carbon phase present in the sample, and its intensity is inversely related to the crystallinity.^41,47,48^

Upon reduction with hydroiodic acid, many changes in Raman spectra are noticed: the 2D peak became clearly distinct, demonstrating the restoration of graphite structure. The D and G_apparent_ peaks become narrower, and D′ becomes blue-shifted, thus becoming less overlapped with the G peak (Figure 8b). The order of D and G_app_ intensity values is: rGO_325_ > rGO_+100_ > rGO_+50_ > rGO_200_; where the intensity values of rGO_325_ and rGO_+100_ are close to each other on one hand, and those of rGO_200_ and rGO_+50_ are close to each other on the other hand. This correlates well with the order of electric conductivities of these films, and is consistent with the intensity-based model of Vecera et al.^46^

The *I*_D_/*I*_G-app_ ratio notably increased after the reduction for all films. However, this does not mean a higher number of structural defects, because according to Ferrari et al.,^40^ a small distance between structural defects (*L*_D_) indicates that rGO is in stage 1, where a strong reduction of sp^3^ is obtained by reduction while a high degree of disorder is caused by the defects left; thus, a higher *I*_D_/*I*_G-app_ ratio in rGO actually indicates a better rGO.^24,26,39^ This ratio is found to increase in our case with the increase of the initial graphite size, together with being quite close for rGO_200_, rGO_+100_, and rGO_+50_ prepared by the enhanced synthesis method. More than having the lowest *I*_D_/*I*_G-app_, the rGO_325_ spectrum is the only one that clearly exhibits D** after reduction, signifying its low order level compared to the other rGO materials. Note that the *I*_D_/*I*_G-app_ ratio is in perfect agreement with the conductivity values, which mirror from one side the presence of reconstructed π bonds and, at low values, the presence of defects, which hinder the electron propagation.

In a trial of quantifying the defects degree, *A*_(all D peaks)_/*A*_G_ was calculated; however, no conclusive data were obtained. The D and D′ bands arise due to the existence of defects such as sp^3^-type, vacancies, edges, and grain boundaries, though their intensities do not always increase linearly with the defect level, because they are induced by a double-resonant Raman mode of graphitic carbon atoms, i.e., activated by the presence of hexagonal rings.^39,45,49^ Thus, their correlation with the defect degree is not fully understood. Nevertheless, the *A*_D′_/*A*_D_ ratio could be used to identify the defects’ nature, where higher values reveal the predominance of vacancy-like defects, and correspond generally to large sheets, while smaller values point out the domination of sp^3^ defects and mostly correspond to small sheets,^43^ which likely fits with the results obtained in this study.

**Figure 9:**
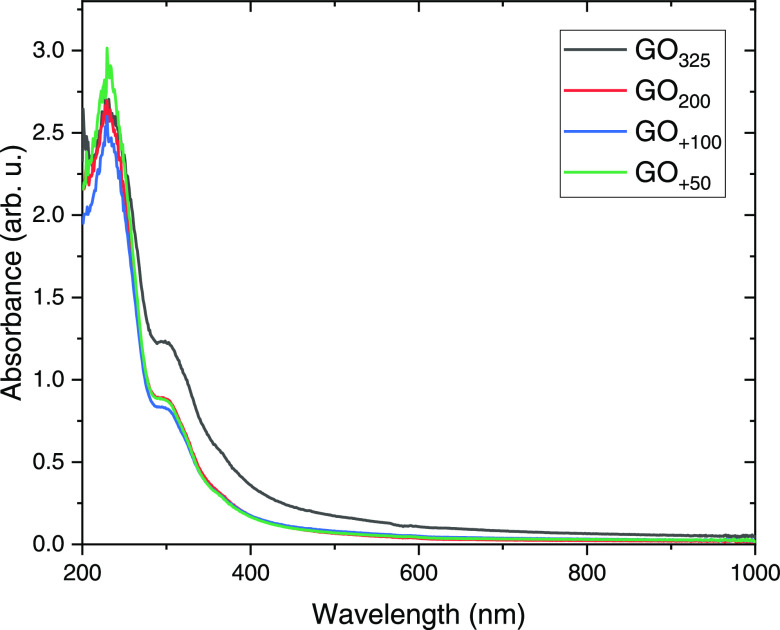
UV/vis spectra of 50 μg/mL GO solutions.

We also investigated the structural differences between the synthesized GO materials using a simple but powerful analytical technique, namely UV–visible spectrometry. The analyzed GO solutions have equal concentrations, and all of their spectra (Figure 9) have a peak at ∼230 nm attributed to the π–π* transition of aromatic C–C bonds, and a shoulder at 300 nm attributed to the *n*–π* transition of the C=O bonds,^50^ suggesting their resemblant structures. GO_+50_ has a slightly higher mass attenuation coefficient (μ) corresponding to the UV light absorbed at λ_π–π*_. So, for an equal amount of each sample, GO_+50_ has likely retained more number of aromatic domains.^28^

**Figure 10:**
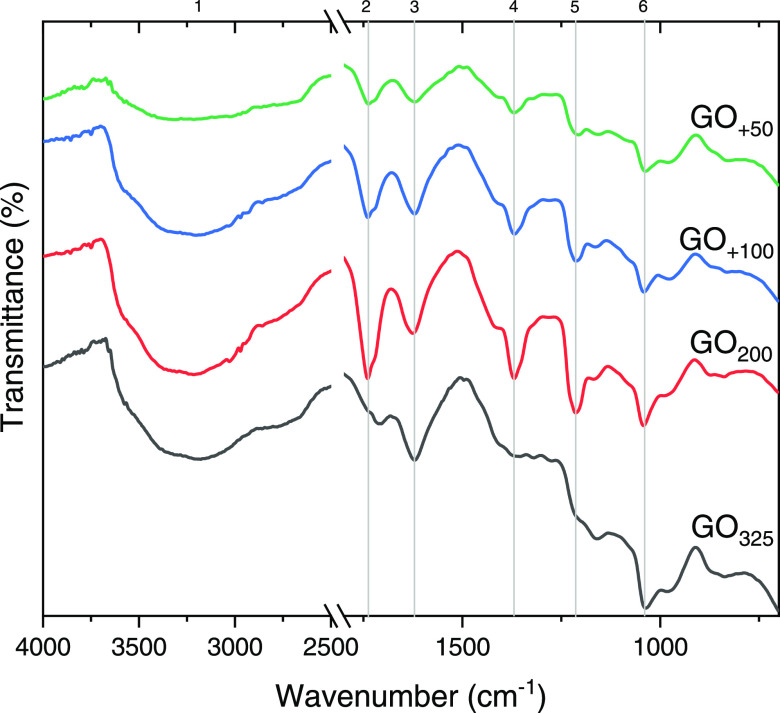
FTIR spectra of GO films.

To study their chemical structures, the different GO films were first analyzed by FTIR spectroscopy. KnowItAll software—academic edition was used to identify the spectra peaks. As shown in Figure 10, all of the GO samples have grossly the same functional groups: (1) the broad O–H stretching vibration (3000–3700 cm^–1^) raised either from the hydroxyl groups in GO or from the adsorbed water molecules; (2) C=O vibration of carbonyl and carboxyl groups manifested at ∼1715–1740 cm^–1^; and (3) the peak at ∼1625 cm^–1^ corresponding to the C=C vibration from sp^2^ bonds in the graphitic plane of GO, in addition to a scissoring mode of water molecules present in the samples.^24^ Finally, OH deformation and C–O stretching vibrations exhibited peaks at ∼1370, ∼1220, and ∼1040 cm^–1^ (peaks number 4–6) corresponding to hydroxyl and epoxy groups. FTIR cannot give a quantitative analysis of the oxidation moieties;^25^ still, some comparisons can be made between the samples. Yet, a careful approach should be followed as GO_+50_ manifested the lowest 3rd peak, which may be due not to low C=C quantity in the sample, but to low water content (more dried sample). This can be confirmed by a lower 1st peak as well. GO_325_ has a red-shifted 2nd peak compared to the other samples, which indicates its high content of carboxyl rather than carbonyl groups. GO_200_, GO_+100_, and GO_+50_ samples manifest more pronounced 4th and 5th peaks compared to GO_325_, which may be due to their high content on C–OH and C–O–C functional groups as will be confirmed by the X-ray photoelectron spectroscopy (XPS) analysis (Figure 12, peak at 286.5 eV).

**Figure 11:**
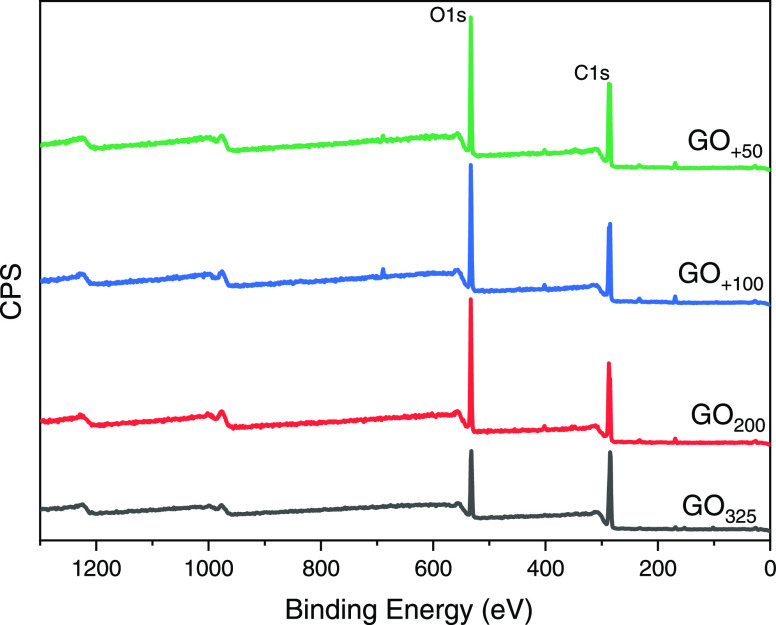
XPS survey spectra of GO films.

Another complementary technique, namely X-ray photoelectron spectroscopy (XPS), also known as electron spectroscopy for chemical analysis (ESCA), was used to determine quantitatively the oxidation degree and the amount of functional groups on each GO material. CasaXPS software was used for processing the data, while Shirley background type was chosen for peak fitting. The C/O ratio was calculated through the survey curves given in Figure 11.

**Figure 12:**
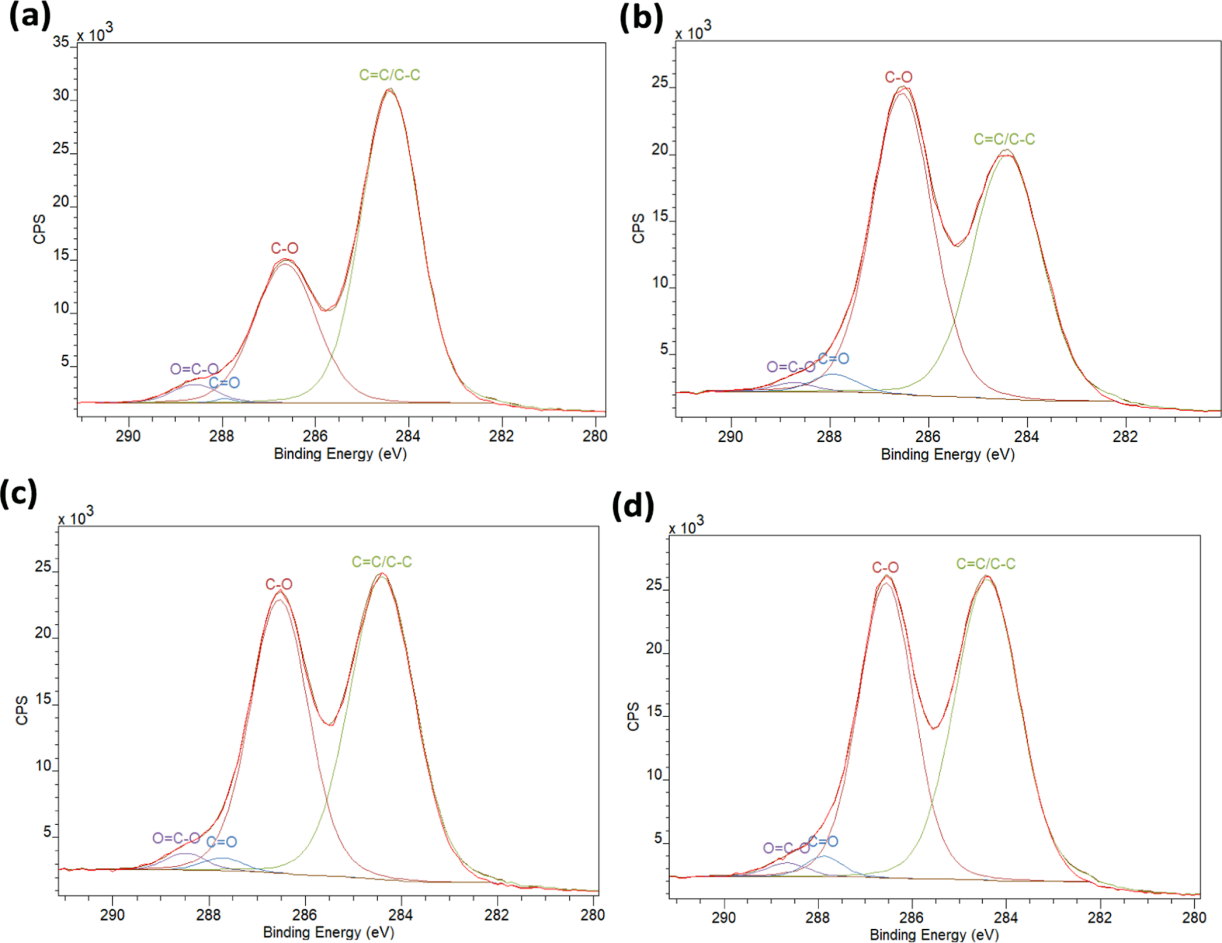
Deconvolution of C 1s XPS peak of (a) GO_325_, (b) GO_200_, (c) GO_+100_, and (d) GO_+50_.

Figure 12 displays the high-resolution XPS spectra of C 1s, which show the bonding of carbon atoms after graphite oxidation, forming either signal (C–O, epoxy, and hydroxyl, ∼286.5 eV) or double bonds (carbonyl and carboxyl groups, 287–289 eV) in all GO films. Minor (<1 eV) shifts in the binding energies were detected between the different GO samples.

Excepting a few impurities found similarly in other works,^25,27^ all samples mainly contain carbon and oxygen (Table S5). GO_200_, GO_+100_, and GO_+50_ have close *C*/*O* values (2.3–2.5), demonstrating similar levels of overall oxidation. However, GO_325_ has a higher *C*/*O* ratio (equal to 3.6), indicating a lower oxidation degree. Despite its low oxidation degree, the deconvolution of C 1s peaks showed that the GO_325_ sample has the highest fraction of carboxyl groups (3% at ∼288.6 eV), as illustrated in Figure 12 and Table S6. According to the Lerf–Klinowski model,^51^ carboxylic groups are formed at the edges of the graphitic sheets, whereas hydroxyl and epoxide groups form on the basal planes of GO sheets. As GO_325_ is produced from small graphite sheets, which have a long edge length for a given area, the resultant GO sheets tend to hold more carboxyl groups. This result is in accordance with the study of Lin et al.^21^ where GO sheets produced from a single graphite source were fractionated into different size groups via three-step centrifugation. Despite the low oxygen content, these carboxylic groups present with a high percentage in GO_325_—compared to the other GOs—are known to reduce the mechanical and electrical properties of the resulting material.^36^ Surprisingly, GO_+100_, which is produced from larger sheets than GO_200_, has a higher fraction of O=C–O (1.8%) compared to GO_200_ and GO_+50_, and also a higher content of impurities, especially organosulfate (2.1 at % sulfur, 5.3 at % of total impurities, Table S6), which may cause more layers’ misalignment,^52,53^ and thus be the reason for the relatively lower mechanical and electrical properties than those of GO_200_, as we have seen.

## 4 Conclusions

This study aimed to interrogate how the initial graphite flake size could influence the physicochemical properties of GO and rGO sheets and freestanding films. Structural and morphological characterization, as well as mechanical and electrical properties were studied using diverse techniques such as scanning electron microscopy (SEM), Raman spectroscopy, Fourier transform infrared spectroscopy (FTIR), X-ray photoelectron spectroscopy (XPS), tensile tests, and the four-point probe technique. This study indicates that the starting graphite size can play a determinant role, but a larger graphite flake size does not always lead to larger and better GO, despite that this trend is ordinarily correct: XPS shows that impurities such as organosulfate, and carboxylic groups located on the sheets’ edges can diminish the properties of the final GO even if it is produced from large graphite flakes. Raman and morphology studies show that as larger graphite flakes need higher oxidant quantity, harsher oxidation occurs to overcome the diffusion-controlled oxidation pathways until arriving at the flakes’ centers, which cuts off the sheets into smaller ones and creates more cracks and defects. Herein, the best GO freestanding film was obtained from +50-mesh graphite and had 232 ± 11 MPa strength and 11.3 ± 1.6 MJ m^–3^ toughness, and its conductivity upon reduction was ∼500 S cm^–1^.

## Supporting Information Available

Images (Figures S1–S6) and Tables (Table S1–S5) giving some experimental details, spectra results and calculations (PDF)
