# Supplementary material for: Graphite Size Effect on Chemical Expansion and Graphene Oxide Properties
Source: ACS Omega. 2022 Oct 14;7(42):37885–95. doi: 10.1021/acsomega.2c05059 (PMC9609075; doi:10.1021/acsomega.2c05059)
Supplement: Supplementary file 1 — ao2c05059_si_001.pdf [file ao2c05059_si_001.pdf]

Supplementary Data

Graphite Size Effect on Chemical Expansion and Graphene Oxide Properties

Zineb Benzait[1], *Levent Trabzon*[1,2,3]

[1] Nanoscience and Nanoengineering Department, Istanbul Technical University, Maslak, Istanbul 34469, Turkey. Email: zineb@itu.edu.tr

[2] Department of Mechanical Engineering, Istanbul Technical University, Istanbul 34437, Turkey

[3] MEMS Research Center, Istanbul Technical University, Istanbul 34437, Turkey

Email: levent.trabzon@itu.edu.tr

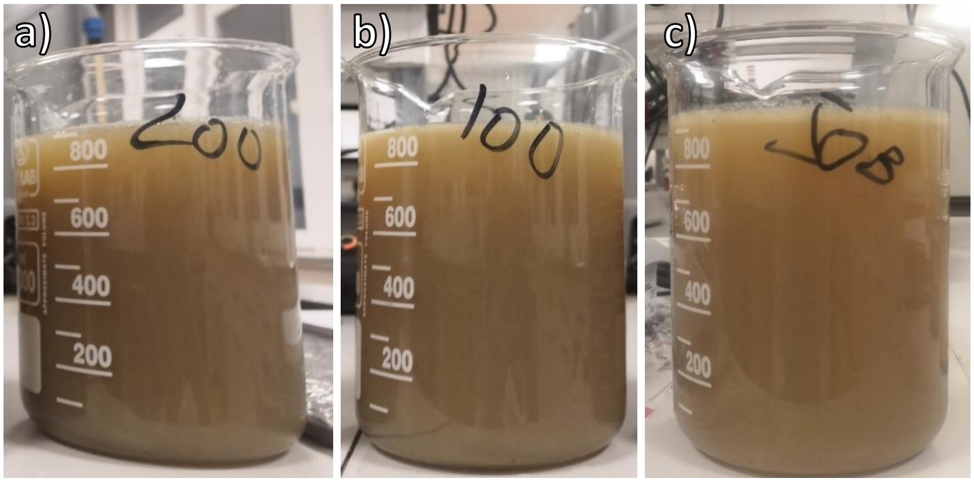

Figure S1. GO prepared by different graphite size (a) 200, (b) +100 and (c) +50 mesh using 2, 3 and 3.5 g of $KMnO_4$ respectively.

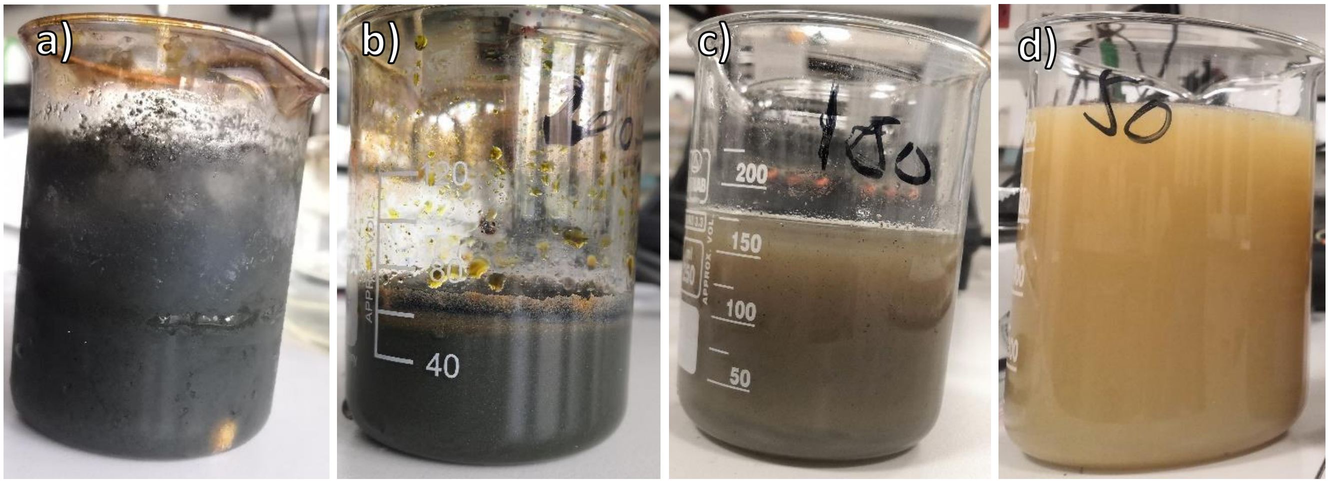

Figure S2. GO prepared using lower (a-c) or higher (d) oxidant quantity.

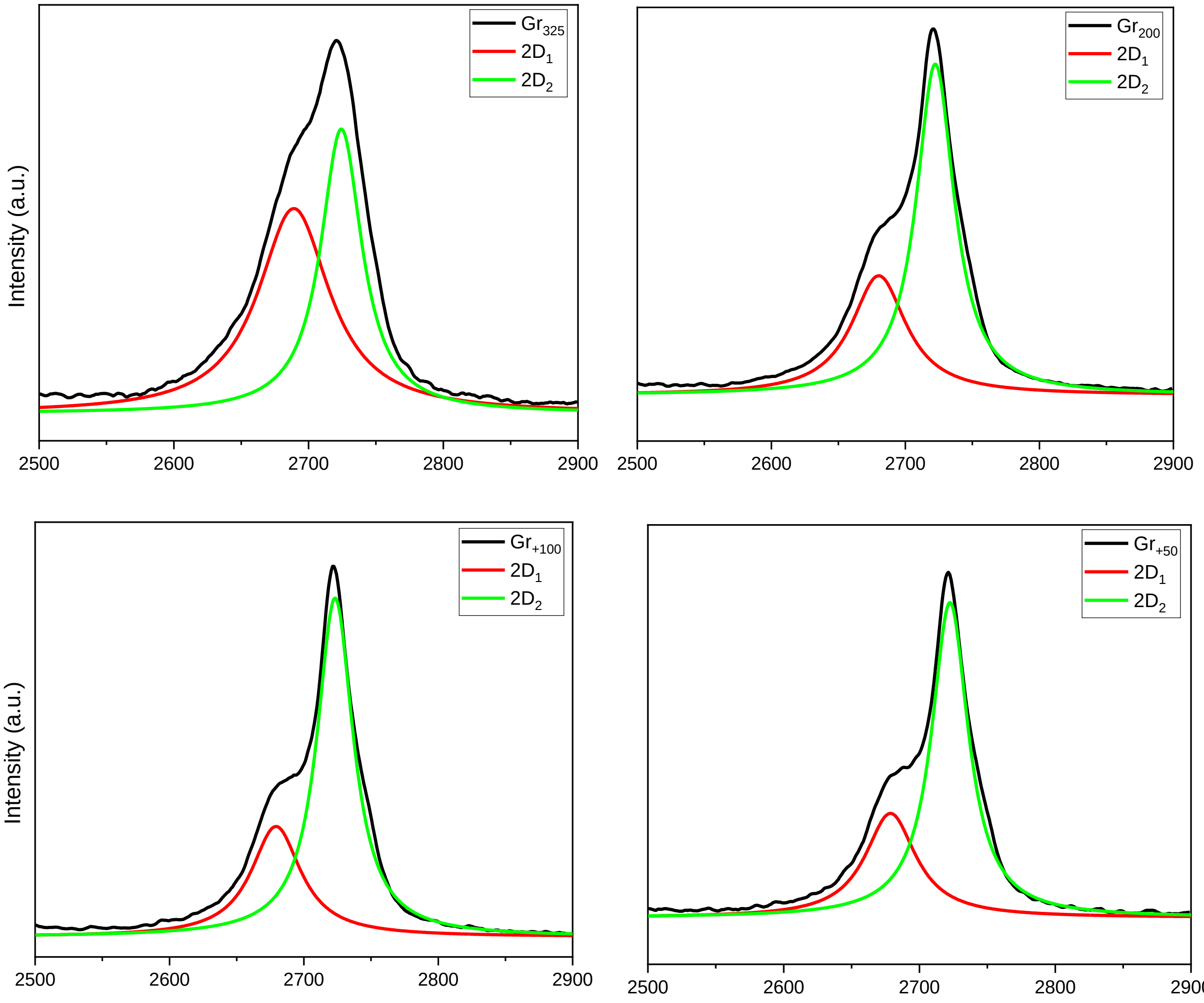

Figure S3. Deconvolution of 2D peak of Raman spectra of graphite specimens.

Table S1. Data derived from the deconvolution of 2D peak of Raman spectra of graphite specimens

	Gr_{325}	Gr_{200}	Gr_{+100}	Gr_{+50}
**I ($2D_2$/$2D_1$)**	1.39	2.77	3.07	3.02
**Position x ($2D_1$)**	2689	2680	2679	2679
**Position x ($2D_2$)**	2724	2722	2723	2722
**x ($2D_2$)-x ($2D_1$)**	35	42	44	44

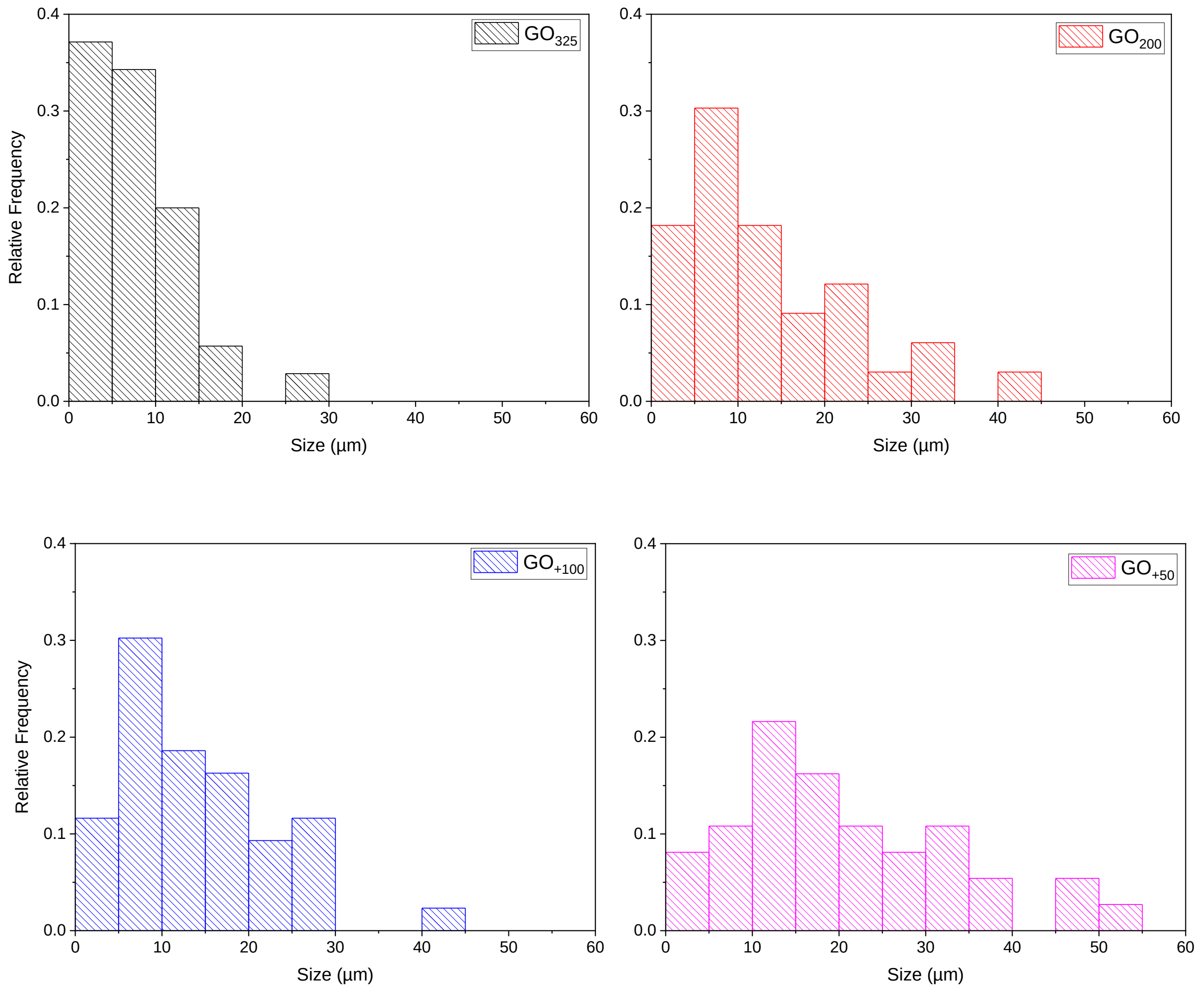

Figure S4. Size distribution histograms of GO sheets (reduced on wafer).

Table S2. Average sheets size and standard deviation of all GO samples, corresponding to the data in Figure S3

Sheets size	GO_{325}	GO_{200}	GO_{+100}	GO_{+50}
Mean	7.5	13.3	14.1	20.7
Standard Deviation	5.2	9.7	8.6	12.4

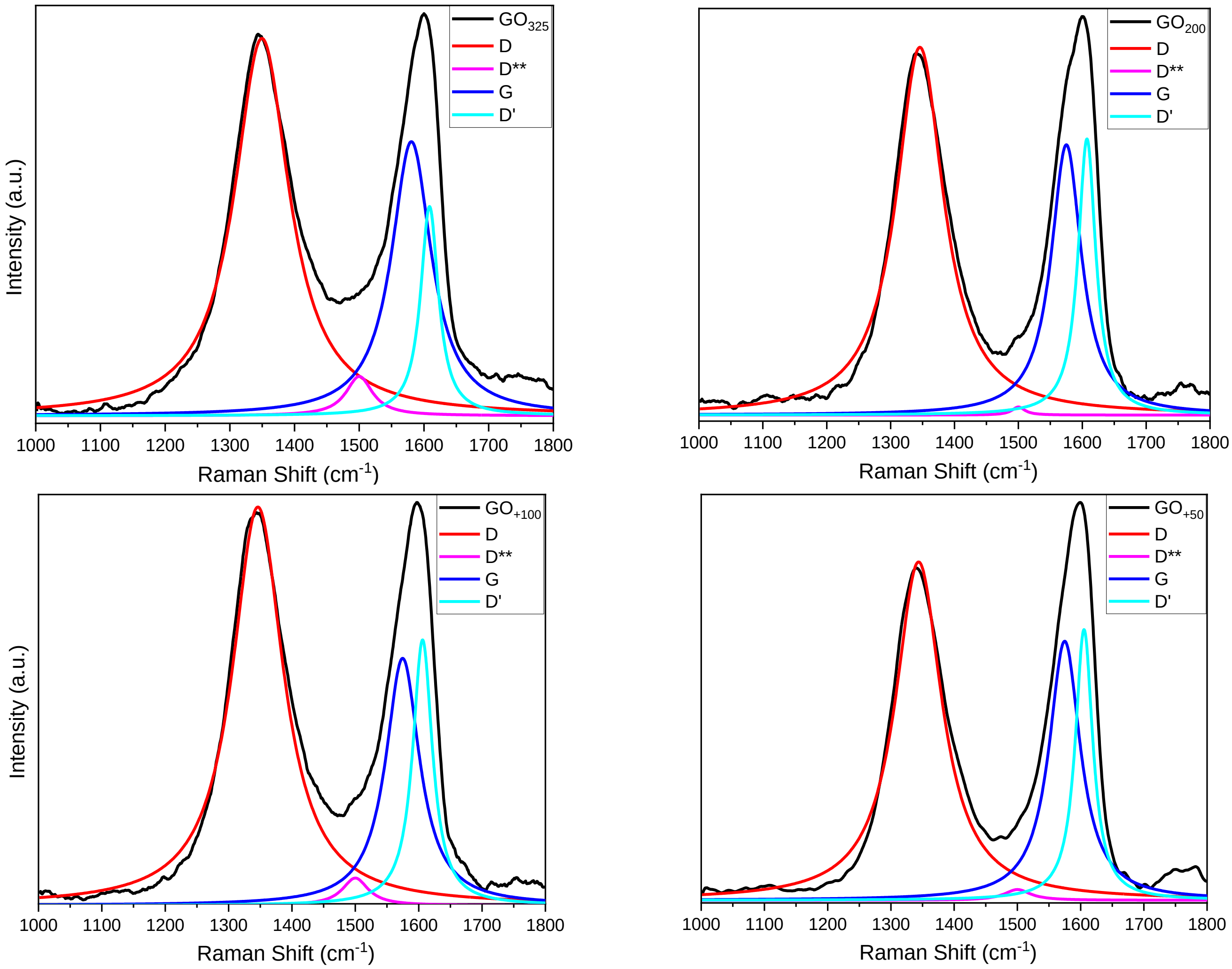

Figure S5. Deconvolution of the main Raman peaks of GO specimens.

Table S3. Data derived from the deconvolution of Raman spectra of GO specimens.

Peak area/Sample	GO_{325}	GO_{200}	GO_{+100}	GO_{+50}
D	150.8	130.1	153.6	119.5
D**	7.6	0.8	5.4	2.1
G	79.3	61.3	63.5	61.7
D'	27.0	36.3	39.4	36.0
$A_{(all\ D\ peaks)}/A_G$	2.3	2.7	3.1	2.6
$A_{D'}/A_D$	0.179	0.279	0.256	0.301
$I_D/I_{G\text{-}app}$	0.95	0.90	0.97	0.83

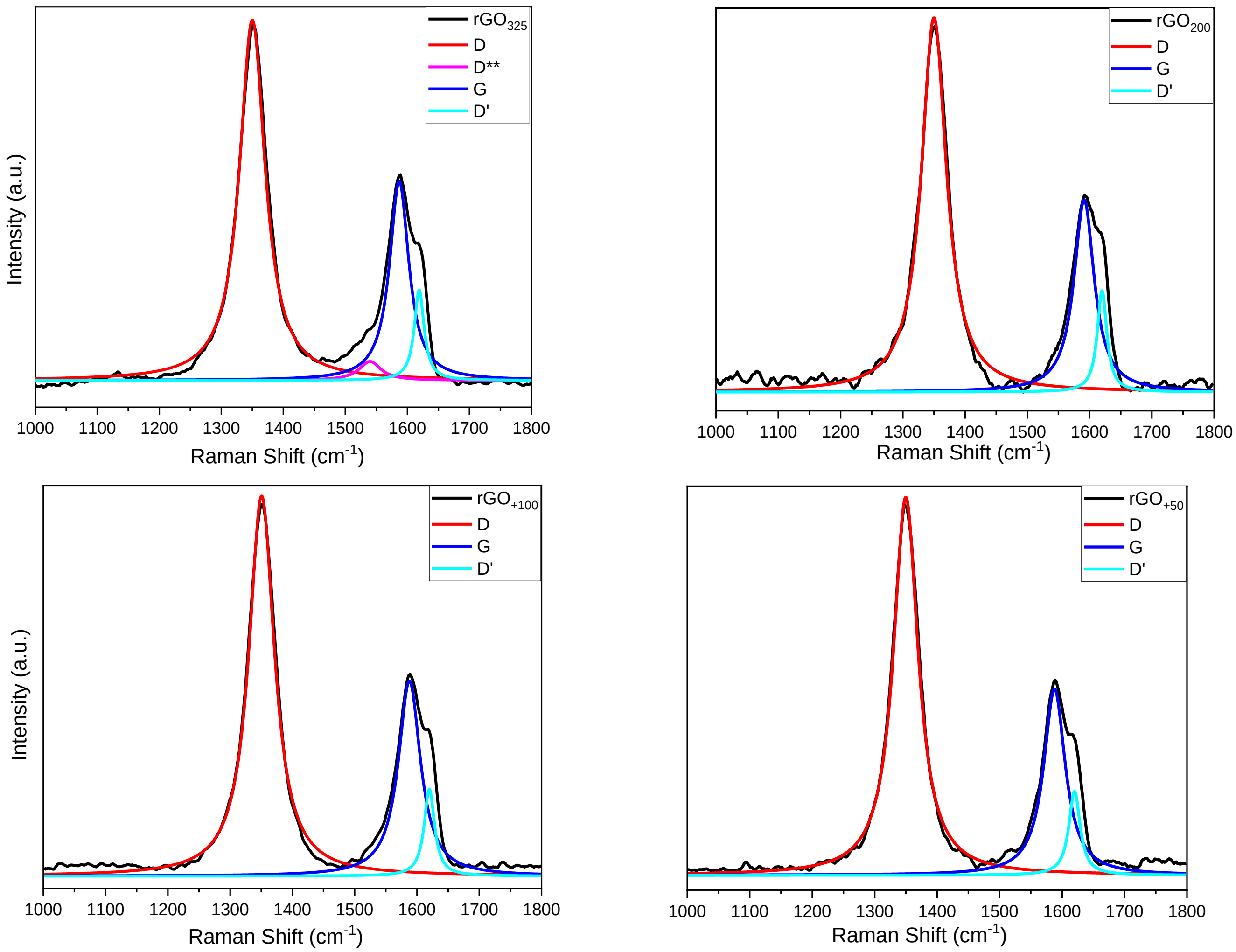

Figure S6. Deconvolution of the main Raman peaks of rGO specimens.

Table S4. Data derived from the deconvolution of Raman spectra of rGO specimens.

Peak area/Sample	rGO_{325}	rGO_{200}	rGO_{+100}	rGO_{+50}
D	77.8	78.5	80.9	77.4
D**	3.5	0.0	0.0	0.0
G	32.4	32.5	36.1	33.4
D'	7.5	8.1	7.6	7.9
$A_{(all\ D\ peaks)}/A_G$	2.7	2.7	2.5	2.6
$A_{D'}/A_D$	0.096	0.103	0.093	0.101
$I_D/I_{G\text{-}app}$	1.71	1.87	1.87	1.92

Table S5. Table of the elemental quantification derived from XPS survey spectra of GO specimens

Sample	GO_{325}	GO_{200}	GO_{+100}	GO_{+50}
C 1s	76.4	67.9	66.5	69.0
O 1s	21.3	29.3	28.3	27.3
N 1s	-	1.6	1.9	1.3
Si 2p	1.2	-	-	-
S 2p	1.1	1.3	2.1	1.6
F 1s	-	-	1.3	0.8

Table S6. Data derived from XPS spectra of GO specimens.

Sample	GO_{325}	GO_{200}	GO_{+100}	GO_{+50}
C/O ratio	3.6	2.3	2.3	2.5
Fraction of C=C/C-C (%)	66.1	48.0	53.7	54.0
Fraction of C-O (%)	30.5	49.1	43.1	43.0
Fraction of O=C-O (%)	3.0	0.9	1.8	1.0
Sulfur content (at%)	1.1	1.3	2.1	1.6
Total impurities content (at%)	2.3	2.9	5.3	3.7
